# Lymphoma of the breast: A mimic of inflammatory breast cancer

**DOI:** 10.1186/1477-7819-9-125

**Published:** 2011-10-11

**Authors:** Nirupama Anne, Ratnakishore Pallapothu

**Affiliations:** 1 Department of Surgery, Our Lady of Lourdes Memorial Hospital, Binghamton, NY, USA

## Abstract

**Background:**

Unusual presentation of breast lymphoma with signs and symptoms suggestive of inflammatory breast cancer.

**Discussion:**

Lymphoma of the breast is uncommon whether it is primary or secondary. Most breast lymphomas are of B cell origin. The most frequent mode of presentation is a painless breast mass. The clinical presentation of localized left breast erythema and edema with an associated left breast mass is common for an inflammatory breast cancer but highly unusual for lymphoma of the breast.

**Conclusion:**

In patients with a left breast mass associated with erythema and edema, the differential diagnosis should include breast lymphoma in addition.

## Background

Lymphoma of the breast is uncommon and it constitutes 0.04% to 0.5% of malignant breast neoplasms [1]. Treatment can include surgery, chemotherapy, and radiation. We report a patient who presented with physical exam mimicking an inflammatory breast cancer however had secondary involvement of the breast by regional lymphoma. The clinical presentation was highly unusual which made the diagnosis a challenge.

## Case Presentation

**Figure 1:**
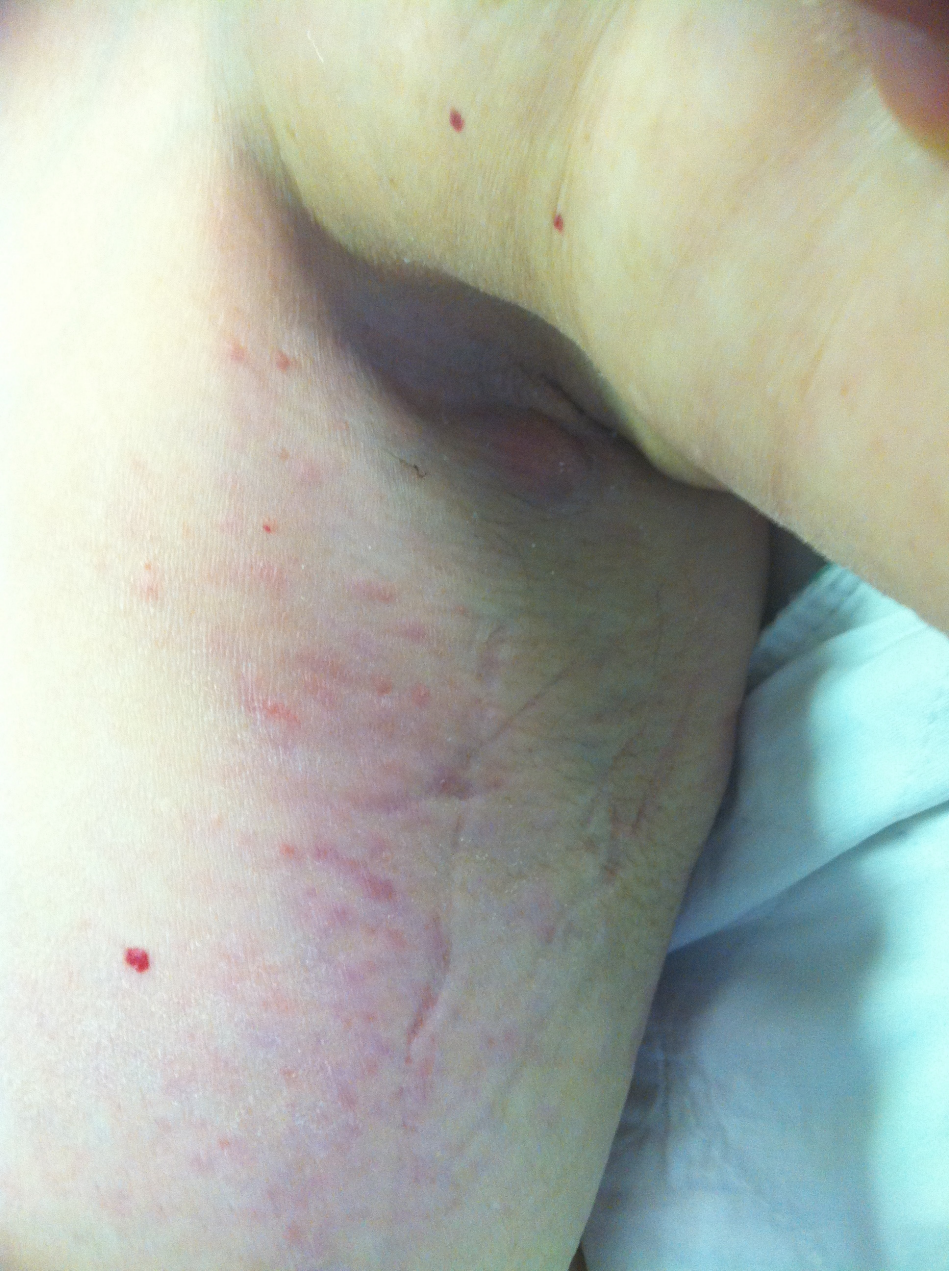
**Image of the extensive erythema and edema**. Image of the extensive erythema and edema of the left breast upper outer quadrant extending into the left axilla.

The patient is an 89 year old Caucasian lady, with multiple medical co-morbidities, who presented to the Emergency Department with decreased functional ability and a history of left breast erythema and edema over one week duration. On exam there was a left breast mass at 12:00, central lesion surrounding the areola, 3 cm × 3 cm, hard, non-tender and with indistinct borders. There was extensive erythema and edema encompassing the upper outer quadrant of the left breast extending into the axilla (Figure 1). The left axilla had palpable, matted lymph nodes, with a superficial 1.5 cm protruding node. Right breast was within normal limits. Examination of the right axilla and supra-clavicular regions revealed fullness. No other cervical or inguinal lymphadenopathy and no organomegaly was noted on abdominal exam.

**Figure 2:**
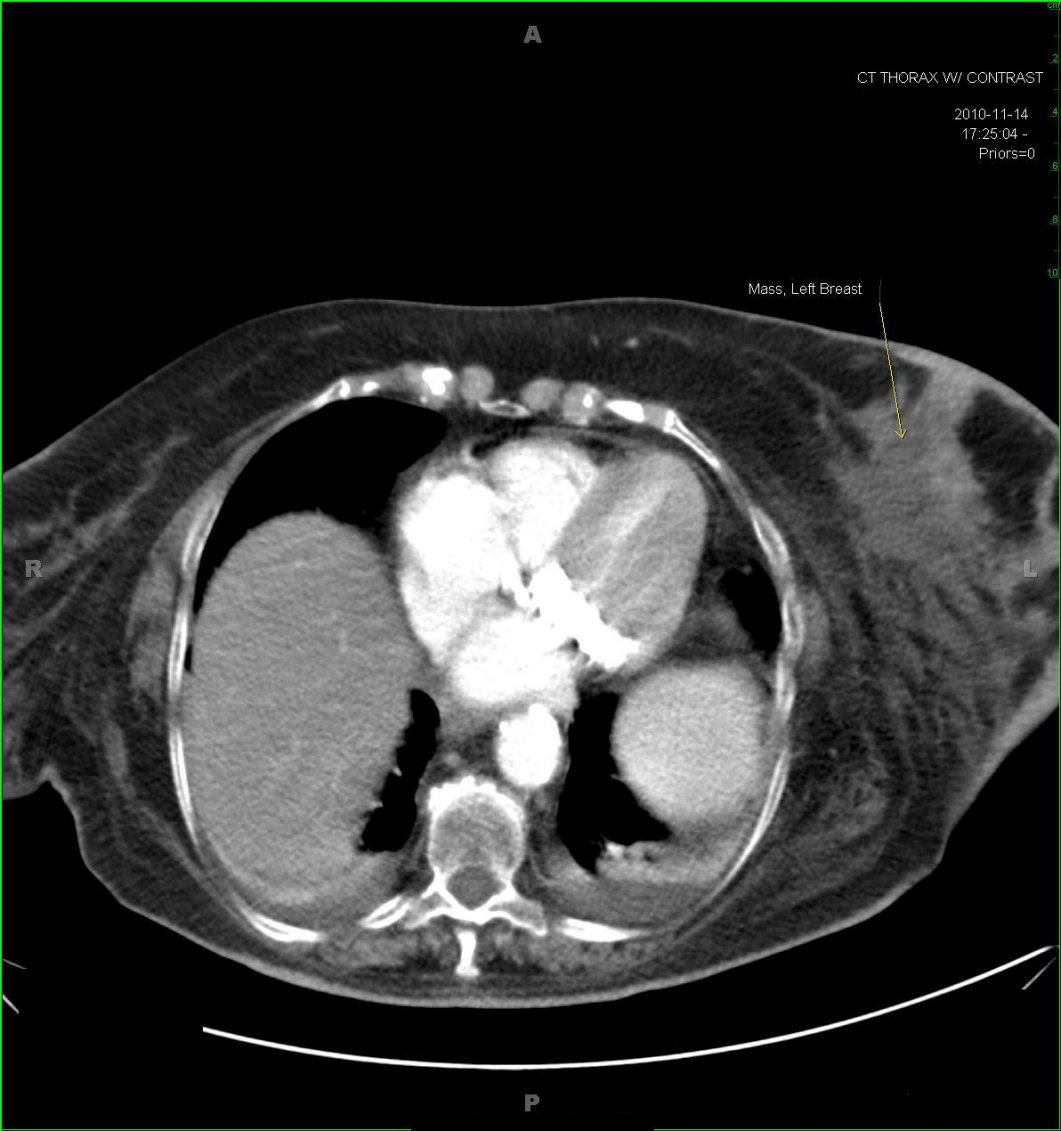
**A CAT scan of the chest**. A CAT scan of the chest demonstrating a large breast mass measuring 7.8 × 7.2 cm in size with associated left breast edema.

Initial laboratory work up showed a normal white blood count of 4,500 with normal differential. A CAT scan of the chest was done which revealed diffuse left breast edema with two large left breast lesions, the largest measuring 7.8 × 7.2 cm in size, (Figure 2) and left axillary adenopathy. It also showed adenopathy involving the right axilla and right supra-clavicular regions. CAT scans of the brain, abdomen and pelvis were normal. She was admitted to the medicine service and treated with intravenous antibiotics based on clinical evidence of left breast erythema/cellulitis.

Breast surgery consultation was obtained. Upon further questioning, the patient admitted to noticing a left axillary mass six months ago, however did not seek any medical attention. She denied constitutional symptoms including fever, chills, night sweats and weight loss. The patient's last mammogram was ten years ago. The patient also has history of dementia.

**Figure 3:**
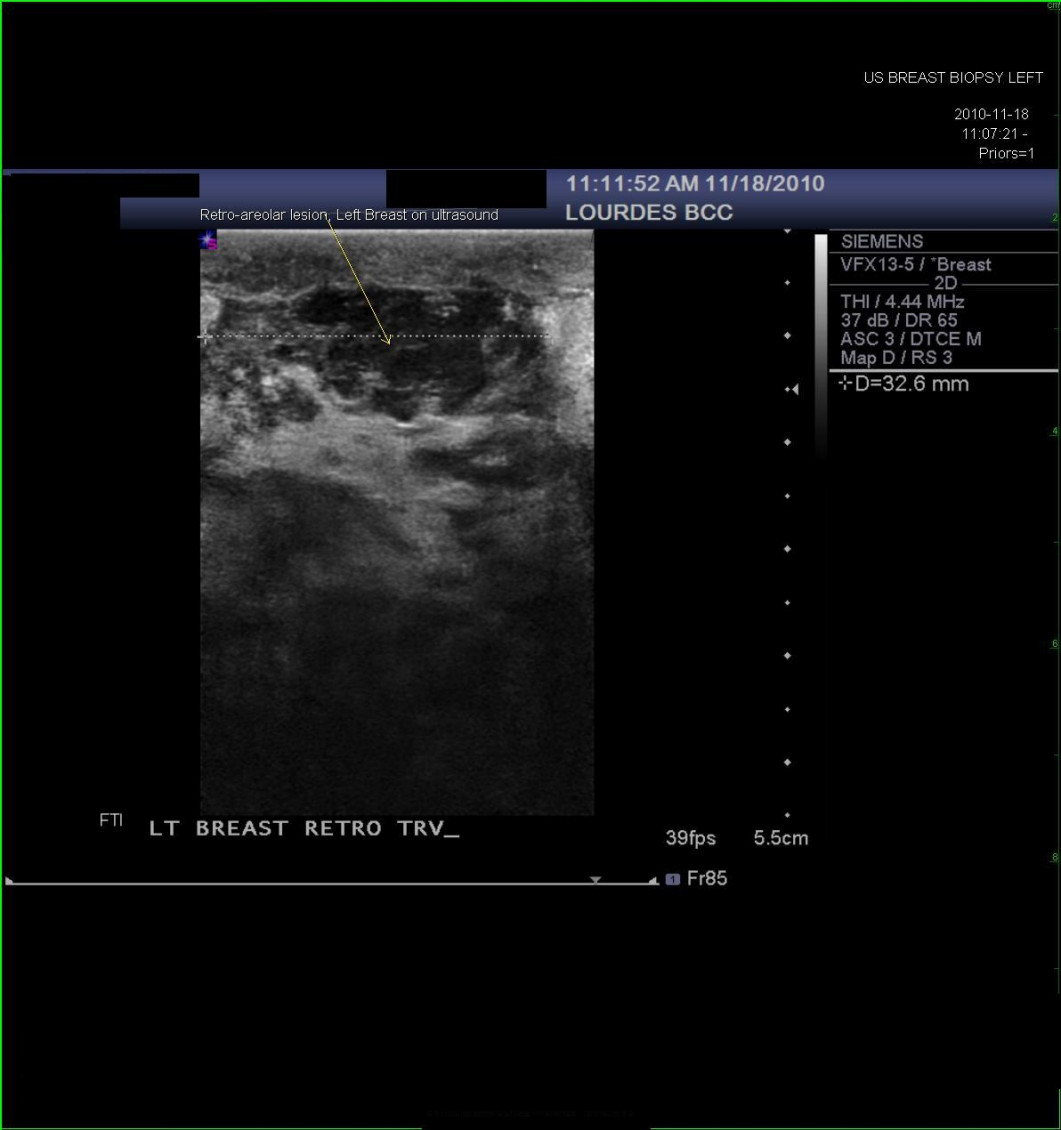
**An ultrasound image of the left breast**. An ultrasound image of the left breast, retro-areolar location, which reveals an ill-defined, hypo-echoic, heterogenous mass corresponding to the palpable mass on physical exam and the larger mass on the CAT scan of the chest.

**Figure 4:**
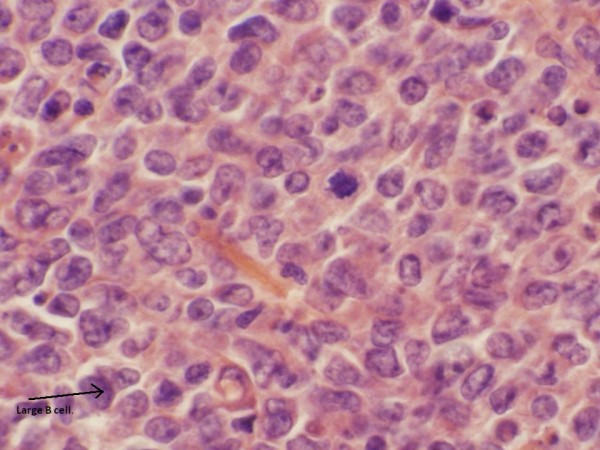
**The slide demonstrates H&E stain of the core biopsy tissue**. The slide demonstrates H&E stain of the core biopsy tissue consistent with a diffuse large B cell lymphoma. The arrow points to a B cell.

**Figure 5:**
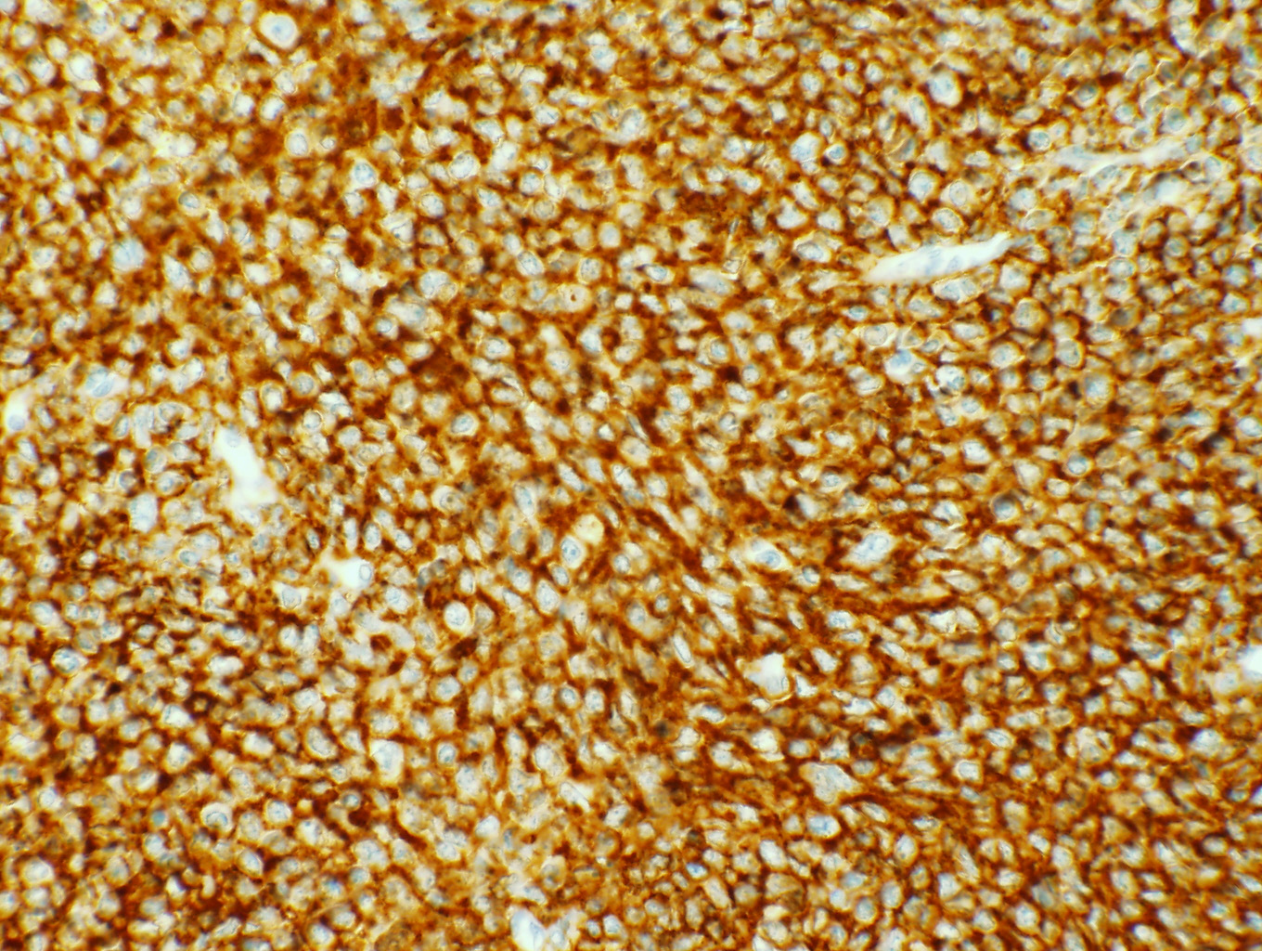
**Immunostain for CD20**. This slide shows that the immunostain for CD20, a B-cell marker, is positive.

The differential diagnosis included lymphoma of the breast as well as inflammatory breast cancer. A diagnostic ultrasound of the left breast was then performed. Ultrasound revealed an ill-defined, retro-areolar, hypoechoic, heterogeneous lesion corresponding to the mass on physical exam and to the larger mass seen on the CAT scan of the chest (Figure 3). The patient subsequently underwent an ultrasound guided left breast core biopsy. Pathology was consistent with diffuse large B cell lymphoma (Figures 4 and 5). She underwent further care with the oncology team.

## Discussion

Lymphoma of the breast is rare either as a primary or a secondary neoplasm. Most breast lymphomas are of B cell origin. The most common histological subtype is a diffuse large B cell lymphoma [2].

In the present case, the patient's initial presentation was with a left axillary mass followed by the left breast erythema and edema and admission to the hospital. On imaging, the tumor was limited to the left breast, left axilla, right axilla, and right supra-clavicular regions, not involving lymphatic chains in other basin. The lymphoma most likely originated in the left axillary lymph node and then involved the left breast as evidenced by the left breast mass on exam and CAT scan findings. Hence we hypothesize that this is a secondary involvement of the breast since there is involvement of the contra-lateral (right) axilla and supra-clavicular regions as well.

Recent literature shows that the incidence of primary lymphoma of the breast might be increasing [3,4]. Primary lymphoma of the breast is defined as the tumor localized to the breast with or without ipsilateral axillary lymph node metastases. In 50% of cases of primary lymphoma, the ipsilateral axillary lymph nodes are involved [5,6].

The presentation of this patient with left breast mass, erythema and edema (Figure 1) is unusual and it does complicate the clinical diagnosis. Most common symptom of breast lymphoma is a painless breast mass, most frequently in the upper outer quadrants [5]. Skin retraction, erythema, peau d' orange appearance, and nipple discharge are uncommon in lymphomas [1-6].

Distinct mammographic or sonographic features have not been described in the literature for breast lymphoma to differentiate it from breast carcinoma or benign breast entities. Imaging with an ultrasound in the present case did not aid in the differential diagnosis of this lesion. The CAT scan of the chest in this case showed lymph node enlargement on the right (contralateral) side which was the key in considering lymphoma in the differential diagnosis of the left breast mass accompanied with erythema and edema which was clinically mimicking inflammatory breast cancer.

The definitive diagnosis should be made with a core biopsy and will allow for therapeutic planning. At present, the treatment of breast lymphoma, whether primary or secondary, should be with systemic chemotherapy [7,8]. The role of surgery is minimal since the tumors are highly sensitive both to chemotherapy and radiation therapy [9-11]. Ryan et al reported statistically significant poor cause-specific survival for patients who underwent mastectomy [12]. Local radiotherapy to involved sites is used to prevent loco-regional recurrence [10-12]. This delivery of radiation therapy alone has been found by Ryan et al to improve overall survival [12]. Kim et al reported that a short course of chemotherapy followed by local radiation therapy was a safe approach for stage IAE disease [11]. For stage IIAE disease, combination of full course of chemotherapy and local radiation therapy has to be considered [11,12]. Survival outcomes were studied in the International Lymphoma Study Group and statistically significant benefit was shown resulting from the addition of radiation therapy to systemic chemotherapy [12].

The Extra Nodal Lymphoma Study Group also did note that young age and stage IIE disease were associated with poor prognosis [12]. Prognosis of patients with breast lymphoma can range from 26 and 66% for 5 year survival rates [13-18]. Diffuse large B cell type has worse prognosis than other histologic subtypes and has significant risk of contralateral breast involvement [13-18]. For secondary lymphoma involving the breast, the prognosis is dependent on the staging of the primary [13-18].

## Conclusions

Lymphoma of the breast is a very rare entity. This case highlights the atypical presentation of lymphoma mimicking an inflammatory breast cancer. Core biopsy of the breast lesion will aid in resolving the diagnostic dilemma and tailor the subsequent management.

## Consent

Written informed consent was obtained from the patient's daughter, health care proxy, for publication of this case report and accompanying images since the patient lacked capacity due to dementia. A copy of the written informed consent is available for review by the Editor-in-Chief of this journal.

## Competing interests

Nirupama Anne: Abstract Selection Committee Member for the American Society of Breast Disease; Regional Speaker for Myriad Genetics Laboratory. No conflict of interest is present with this case report. Ratnakishore Pallapothu: None.

## Authors' contributions

NA: Literature review, collection of case report, writing, and editing the manuscript. RP: Literature review, writing, and editing the manuscript. Both authors read and approved the final manuscript.
